# Emerging Global Patterns of Terbinafine Resistance in *Trichophyton* Species: A Systematic Review and Meta-Analysis

**DOI:** 10.3390/antibiotics15070699

**Published:** 2026-07-17

**Authors:** Imdat Kilbas, Elmas Pinar Kahraman Kilbas, Ihsan Hakki Ciftci, Norberth-Istvan Varga, Florin George Horhat

**Affiliations:** 1 Dental Prosthesis Technology Program, Vocational School of Health Services, Fenerbahce University, Istanbul 34758, Türkiye; imdtklbs@gmail.com; 2 Medical Laboratory Techniques Program, Vocational School of Health Services, Fenerbahce University, Istanbul 34758, Türkiye; elmspnrkk@gmail.com; 3 Department of Medical Microbiology, Faculty of Medicine, Sakarya University, Sakarya 54100, Türkiye; 4 Multidisciplinary Research Center on Antimicrobial Resistance (MULTI-REZ), Microbiology Department, Victor Babes University of Medicine and Pharmacy, 300041 Timisoara, Romania; 5 Department of Microbiology, Victor Babes University of Medicine and Pharmacy, Eftimie Murgu Square No. 2, 300041 Timisoara, Romania

**Keywords:** *Trichophyton*, terbinafine, drug resistance, antifungal agents, prevalence

## Abstract

**Background/Objectives**: In recent years, reports regarding terbinafine resistance in *Trichophyton* spp. have increased. The aim of this study is to evaluate the prevalence of terbinafine resistance in *Trichophyton* spp. species on a regional and species basis and to systematically analyze the antifungal susceptibility testing methods used and the available data. **Methods:** This systematic review and meta-analysis was conducted in accordance with the PRISMA guideline. Studies published until 1 December 2025 were searched in the PubMed/MEDLINE, Scopus, Web of Science, and Embase databases. Studies reporting antifungal susceptibility test results using species-level identification, standardized, or study-defined methodologies were included. Pooled prevalence estimates were calculated with a random-effects model. Heterogeneity was evaluated with the I^2^ statistic. Subgroup analyses were performed according to publication period, resistance definition groups, species, and geographic region. *SQLE* mutation patterns were summarized descriptively. **Results:** Nineteen studies reported from 13 different countries were included in the meta-analysis. In sensitivity analyses that included surveillance-based studies, the combined prevalence rate was found to be 3.2% (95% confidence interval 1.5–6.7). Higher descriptive resistance levels were observed in *T. indotineae* and *T. mentagrophytes*, although species-related differences were not statistically significant. Among studies reporting *SQLE* mutations, alterations involving codons 393 and 397 were the most frequently described; however, mutation patterns were summarized descriptively because of methodological heterogeneity among studies. **Conclusions:** *Trichophyton* spp. terbinafine resistance is an emerging global threat. Standardized susceptibility testing, harmonized epidemiological cut-off values, and enhanced molecular surveillance are essential for accurate detection, resistance monitoring, and effective clinical management.

## 1. Introduction

In recent years, globalization and international migration have increased the spread of microorganisms and infectious diseases, including fungal pathogens and the diseases associated with them [1,2]. Particularly antifungal-resistant fungal infections constitute an increasingly important public health threat worldwide [3,4]. The widespread or long-term use of broad-spectrum antifungals, use of invasive medical devices, prolonged hospital stays, immunosuppression, surgical interventions, or hemodialysis are factors that predispose to fungal infections. This situation creates a significant clinical burden and economic cost on healthcare systems [4].

Dermatophytoses are the most common fungal infections worldwide and affect approximately one quarter of the global population [5]. Dermatophytosis is a superficial fungal infection that selectively involves keratin-rich structures (stratum corneum, hair and nails) and causes ring-shaped lesions and alopecic areas. Although they are generally considered mild infections, due to their high contagiousness they can significantly reduce patients’ quality of life by causing itching, burning sensation, sleep disorders, depression, and stigmatization [6]. These infections have traditionally been named by adding the Latin term tinea, which defines the affected anatomical region (for example, tinea capitis) [4].

In identifications made using molecular methods, it has been shown that the genera *Trichophyton*, *Epidermophyton*, *Nannizzia*, *Paraphyton*, *Lophophyton*, *Microsporum* and *Arthroderma* play a role as causative agents of infection [7].

One of the most commonly used systemic antifungal agents in the treatment of dermatophyte infections is terbinafine. Terbinafine, an allylamine group antifungal agent, was developed by Sandoz in 1984 and has since been used worldwide in the treatment of fungal infections. Terbinafine inhibits the squalene epoxidase enzyme involved in the ergosterol biosynthesis pathway, preventing the conversion of squalene to oxidosqualene. This leads to ergosterol deficiency, suppresses fungal growth, and induces cell death [4,8,9].

Treatment with terbinafine or itraconazole can be effective in the eradication of infections caused by *Trichophyton* spp. [10]. However, point mutations occurring in the squalene epoxidase (*SQLE*) gene can lead to the emergence of resistant forms and reduce treatment success. In addition, although different methods are available, antifungal susceptibility tests for dermatophytes are not routinely performed and cut-off values (clinical breakpoints) that allow classification of isolates as susceptible or resistant have not yet been defined [4].

Terbinafine-resistant *Trichophyton rubrum* strains were first identified in 2003 in North America in a patient who received terbinafine treatment for 24 months [11]. Subsequently, in 2006, another terbinafine-resistant *T. rubrum* case was reported from the same country [12]. Despite numerous important studies published since 2017, an increase has been observed globally in the number of terbinafine-resistant strains of *T. rubrum*, *T. interdigitale*, *T. mentagrophytes* and *T. indotineae*; this situation has prompted extensive research into the underlying resistance mechanisms [13,14,15,16].

Treatment failure in dermatophytosis is associated with insufficient treatment duration, inability to eliminate predisposing factors, reinfection, and increasing antifungal resistance. For effective treatment, correct identification of the causative agent at species level and application of in vitro antifungal susceptibility tests are important. The aim of this study is to reveal the regional trends of terbinafine resistance prevalence in *Trichophyton* spp. species, to evaluate resistance rates according to species, and to systematically analyze the available data by taking into account the differences between the antifungal susceptibility test methods used and the guidelines.

## 2. Results

### 2.1. Study Characteristics

**Figure 1:**
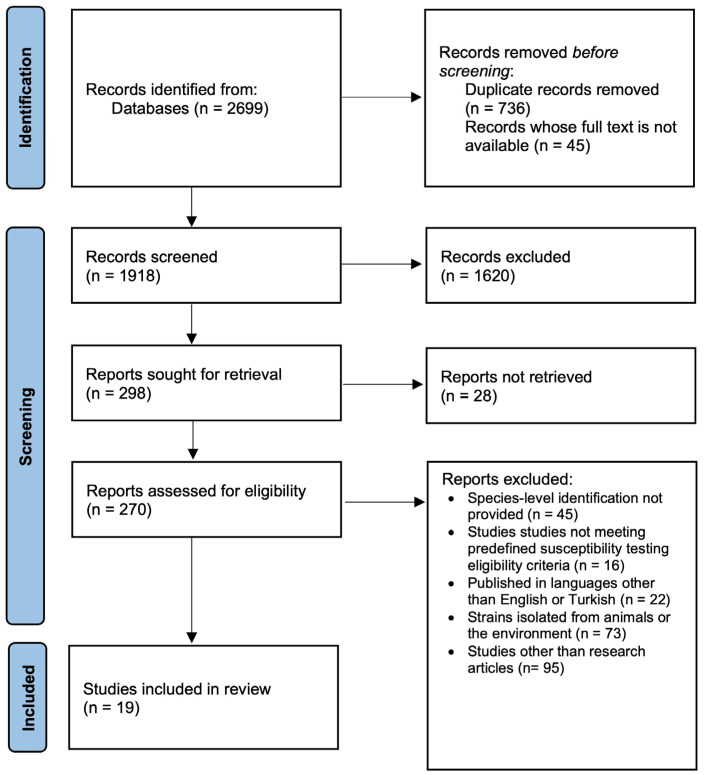
Studies were excluded and included based on the search criteria (PRISMA flow chart).

The total number of studies found according to the keywords identified in the databases was 2699, and the full texts of 298 studies were accessed. After evaluation according to the exclusion criteria, a total of 19 original research articles were included in the study (Figure 1). The large number of excluded records reflects the predefined eligibility criteria focusing on MIC-based antifungal susceptibility testing, species-level identification, inclusion of human clinical isolates, and original research articles, ensuring methodological consistency across the included studies.

The 19 included studies were reported from 13 different countries and comprise a total of 4111 dermatophyte isolates. In the current literature, terbinafine resistance in dermatophytes was investigated. However, no terbinafine resistance data were identified for other dermatophyte genera such as *Microsporum* or *Epidermophyton*. Terbinafine resistance rates of *T. rubrum* were reported in nine studies, *T. indotineae* in five studies, *T. interdigitale* in seven studies, and *T. mentagrophytes* in four studies. The main characteristics of the studies are summarized in Table 1. 

### 2.2. Reported SQLE Mutation Patterns

*SQLE* mutations associated with terbinafine resistance were reported in 14 of the included studies. Across these studies, the most frequently reported mutations involved codons 393 and 397, particularly the L393F/L393S and F397L substitutions. However, direct quantitative comparison of mutation frequencies across studies was not considered appropriate because the included studies differed substantially with respect to the Trichophyton species investigated, the molecular methods used (targeted hotspot PCR versus full-gene sequencing), and the analyzed *SQLE* regions. Therefore, mutation patterns are presented descriptively rather than quantitatively.

Due to heterogeneity, both fixed-effect and random-effects models were evaluated in the meta-analysis. In the fixed-effect model, the pooled prevalence of terbinafine resistance was estimated at 6.0% (95% CI: 5.0–7.1%). However, significant heterogeneity was observed among studies (Q = 135.09, df = 9, *p* < 0.001; I^2^ = 93.34%). Therefore, the random-effects model was considered more appropriate. Using the random-effects model, the pooled prevalence of terbinafine resistance was estimated at 3.2% (95% CI: 1.5–6.7%). Although the pooled estimate decreased substantially after restricting the analysis to surveillance-based studies (Groups 1 and 2), the wide confidence interval and high I^2^ value indicate considerable residual heterogeneity among the included studies (Table 2).

**Figure 2:**
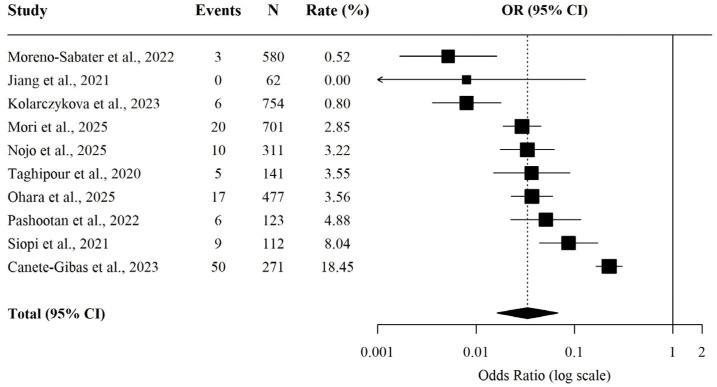
Forest plot of terbinafine resistance prevalence in surveillance-based studies (Groups 1 and 2 only) [8,17,18,19,20,21,22,23,24,25].

**Figure A1:**
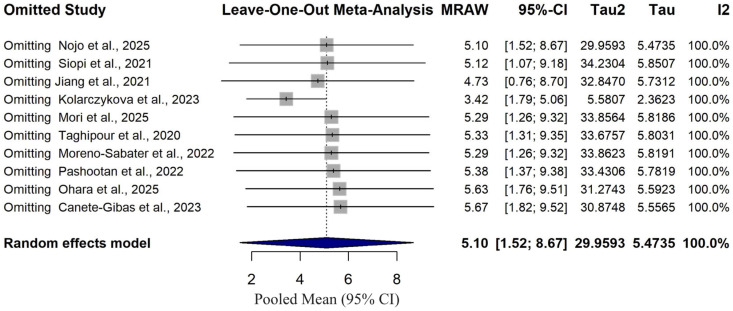
Leave-one-out sensitivity analysis for surveillance-based studies (Groups 1 and 2) [8,17,18,19,20,21,22,23,24,25].

As shown in the forest plot, terbinafine resistance rates in surveillance-based studies (Groups 1 and 2) ranged from 0% to 18.5% (Figure 2). When Group 3 studies, which included resistant referrals, reference collections, or highly selected populations, were excluded, the pooled prevalence estimate decreased from 8.6% to 3.2%. This indicates that the overall estimate was influenced by studies enriched for resistant isolates. Although substantial heterogeneity remained (I^2^ = 93.3%), leave-one-out sensitivity analyses demonstrated that exclusion of any single study did not materially alter the pooled prevalence estimate (Figure A1), indicating that no individual study exerted a disproportionate influence on the overall findings.

### 2.3. Subgroup Analyses

Mean rank values were higher in studies published in 2022 and later (11.13) compared to studies published in 2021 and earlier (5.75). However, no statistically significant difference was observed in the prevalence of terbinafine resistance between studies published in 2021 and before and those published in 2022 and after (Mann–Whitney U = 13.0, Z = −1.703, *p* = 0.089).

Terbinafine resistance did not show a statistically significant difference among study groups defined according to the resistance assessment approach (as explained above; group 1, group 2, group 3) (Kruskal–Wallis H = 4.785, df = 2, *p* = 0.091). Mean rank values were highest in group 3 (12.67), followed by group 1 (9.00) and lowest in group 2 (5.50).

Species classification was done according to the descriptions reported in the original studies. In some studies, isolates were specified as *T. mentagrophytes* without further differentiation within the species complex, and because retrospective reassignment to *T. indotineae* or other lineages was not done. Among species, mean rank values for terbinafine resistance from highest to lowest were determined as *T. indotineae* (17.00) and *T. mentagrophytes* (16.00), followed by *T. rubrum* (11.61) and *T. interdigitale* (10.21). However, no statistically significant difference was detected in terms of terbinafine resistance prevalence among *Trichophyton* species (Kruskal–Wallis H = 3.582, df = 3, *p* = 0.310) (Table A3). These findings suggest that observed variations are descriptive and the pooled prevalence should be interpreted as an indicator of resistance trends rather than exact global prevalence.

No statistically significant difference was detected in terbinafine resistance prevalence among geographic regions in *Trichophyton* species (Kruskal–Wallis H = 3.908, df = 2, *p* = 0.142). Prevalence comparisons by region were evaluated as secondary and descriptive analyses to examine possible sources of heterogeneity. At this stage, studies in group 3 were not excluded from the main meta-analysis, but were handled separately to evaluate the patterns of surveillance-based studies. As a result of the evaluation, it was observed that in the secondary analyses focusing on surveillance-based studies, no study representing South Asian countries remained, and North America was represented by only a single study (terbinafine resistance rate 18.45%). This regional representation, particularly due to the single study reporting a high resistance rate, affected the rank-based measures used for regional comparison and limited the interpretation of geographic resistance prevalence. Therefore, findings related to geographic regions were evaluated at a descriptive level rather than for statistical inference purposes.

## 3. Discussion

This study demonstrates a growing number of reports of terbinafin resistance in *Trichophyton* species worldwide. However, the resulting aggregate estimate should be interpreted as a synthesis across heterogeneous study designs and populations, rather than as a single stable global prevalence value. The different antifungal susceptibility testing methods used in the included studies and variable MIC thresholds are considered to be one of the major contributors for the high heterogeneity observed (i^2^ = 93.3%).

In recent years, *T. rubrum* has been reported as the most common causative organism of dermatophytosis [33]. In the studies included in the meta-analysis, the three most common agents were *T. rubrum*, *T. indotineae*, and *T. interdigitale*, respectively. Today, *T. indotineae*, which is frequently resistant to terbinafine, has led to the spread of dermatophytosis cases that are chronic, recurrent, and may cause treatment failure, particularly in the Indian subcontinent [34]. According to some studies, it has been reported to exhibit high in vitro minimum inhibitory concentrations (MIC ≥ 1 μg/mL) with a prevalence of up to 75%. The proportion of terbinafine-resistant *T. indotineae* isolates with terbinafine MIC values ≥16 μg/mL was 90% in the study by Bidaud et al. (2023) and 17% in the study by Rhodes et al. (2026) [30,35]. Although high MIC values in in vitro tests have been associated with treatment failure, it is reported that the relationship between phenotypic resistance and clinical outcome is not always significant [36]. The marked heterogeneity found among studies in this meta-analysis suggests that in vitro resistance rates alone may be insufficient to explain the clinical course. Variability in MIC thresholds, test methods, and patient populations in dermatophytes complicates the correlation of laboratory-based resistance findings with clinical response. In addition, due to the lack of clinical breakpoints for dermatophytes, the use of epidemiological cut-off values, although allowing differentiation of wild-type and non-wild-type isolates, should be considered not to directly indicate clinical failure. Therefore, integrating phenotypic susceptibility tests with molecular data and clinical response may strengthen the clinical meaning of laboratory-based resistance definitions.

The absence of globally accepted clinical breakpoints for dermatophytes makes resistance definitions based on fixed MIC thresholds difficult. In this context, demonstrating *SQLE* mutations associated with terbinafine resistance, particularly in *T. indotineae* strains, supports resistance detection [37]. Terbinafine resistance causes treatment failure and infection relapse in dermatophytosis [38]. Terbinafine inhibits the squalene epoxidase enzyme that converts squalene to 2,3-oxidosqualene. Thus, squalene, which is toxic for fungal cells, accumulates in the cell. Structural changes in squalene epoxidase may weaken its interaction with terbinafine while preserving its essential function in ergosterol biosynthesis [39]. Across the included studies, mutations involving codons 393 and 397 were among the most frequently reported. However, because the studies differed considerably with respect to the Trichophyton species examined, the molecular methods employed (targeted hotspot PCR versus full *SQLE* gene sequencing), and the extent of *SQLE* sequencing, direct quantitative comparison of mutation frequencies was not considered appropriate. Consequently, these findings should be interpreted descriptively rather than as evidence of the true global distribution of *SQLE* mutations. However, the absence of *SQLE* mutations in some phenotypically terbinafine-resistant isolates suggests that additional molecular mechanisms may play a role in resistance. Studies in which EUCAST-based screening methods are supported by molecular confirmation may help to correctly identify resistant strains as well as optimize resource use. Our findings indicate that terbinafine resistance in dermatophytes should be addressed by evaluating molecular tests and clinical condition together in addition to in vitro MIC determination, and also reveal an urgent need for standardized and accessible resistance monitoring strategies at national and global levels.

Molecular and phenotypic findings suggest the possibility of international spread of closely related resistant lineages, particularly among *T. indotineae* strains. In addition, reports of increased MIC values to alternative antifungal drugs such as miconazole and griseofulvin may indicate a trend towards decreasing susceptibility; however, more standardized studies are needed to definitively determine multidrug resistance [40]. The findings obtained in this meta-analysis are conceptually consistent with previously reported trends of higher terbinafine resistance in *T. indotineae*. However, no statistically significant difference was detected among species in the present analysis. Therefore, the higher rank values observed for *T. indotineae* and the high MIC values reported in some studies should be interpreted as descriptive observations rather than definitive species-specific differences. In addition to transmission of dermatophytes among humans, the widespread and inappropriate use of steroid-containing antifungal creams has been proposed as a potential factor contributing to selection pressure in dermatophyte populations, but the exact causal relationships have not yet been fully established. The acceleration of global migration and travel movements has facilitated the geographic spread of resistant clones and transformed dermatophytosis from an endemic problem into an international public health issue.

This study included data from 13 different countries and showed that terbinafine resistance in dermatophytes has become a significant global concern, with a pooled resistance prevalence of 8.6% based on a random-effects model. Exclusion of Group 3 studies reduced the pooled prevalence from 8.6% to 3.2%. As shown in Figure 2, heterogeneity remains high (I^2^ = 93.3%), partly due to differences in resistance definitions across studies. Given the significant heterogeneity in the studies and the inclusion of studies from suspected or referral populations, the combined prevalence estimate should be interpreted as an indicator of the presence and range of resistance, rather than a precise measure of global prevalence. In previous reports, particularly high rates of terbinafine resistance in *T. indotineae* species have been reported in South Asia, and this region has been defined as a focal point in terms of resistance [41]. The absence of a significant difference in resistance rates according to geographic regions in this meta-analysis suggests that findings regarding regional distribution may have been affected by study design and representation imbalance. In the secondary evaluation based on surveillance-based studies, the absence of a study representing South Asia and the representation of North America by only a single study limited the interpretation of regional comparisons, particularly due to the high resistance rate reported in this study. However, terbinafine-resistant dermatophyte infections are not limited to South Asia, and strains harboring the *SQLE* phenylalanine-leucine amino acid substitution (F397L) at codon 397, together with other strains causing terbinafine resistance, have been detected in Russia, Japan, Greece, Iran, Switzerland, France, Belgium, Finland, Germany, Denmark, Poland, the United States, and Canada [41,42,43,44]. Therefore, the absence of a significant difference according to geographic regions may be considered both a reflection of regional representation imbalances and a finding consistent with the spread of resistant strains to different geographies, particularly after 2021.

An important limitation of the included studies is the frequent absence of genotyping or sequence-based species confirmation. In many studies, isolates within the *T. mentagrophytes* complex were reported without further molecular differentiation, and retrospective reclassification into *T. indotineae* or related lineages was not performed. This may lead to misclassification bias, particularly in studies conducted before the formal recognition of *T. indotineae* in 2020.

An additional limitation of this meta-analysis is the lack of standardized definitions of terbinafine resistance across included studies. Some studies applied CLSI breakpoints, others used EUCAST ECOFFs, and a few used non standardized laboratory criteria. Consequently, the term “resistance prevalence” may not represent the same biological phenomenon in all studies, which likely contributes to the observed heterogeneity. The combined prevalence should be interpreted as an approximate indicator of resistance trends, rather than a precise measure of global resistance.

These methodological constraints are common across dermatophyte resistance studies and may contribute to between-study heterogeneity in species identification and susceptibility testing approaches.

## 4. Materials and Methods

### 4.1. Study Design

This study is a systematic review and meta-analysis conducted to evaluate the prevalence of terbinafine resistance in dermatophytes. The study design was based on the internationally recognized PRISMA (Preferred Reporting Items for Systematic Reviews and Meta-Analyses) guidelines for systematic reviews and meta-analyses [45] and was registered in the PROSPERO database under the number CRD420261281356. The PRISMA checklist is presented in Table A1.

### 4.2. Literature Search Strategy

The systematic literature search was conducted in the PubMed/MEDLINE, Scopus, Web of Science, and Embase databases for studies published until 1 December 2025. The search was performed using the keywords “dermatophyte”, “*Trichophyton*”, “*Trichophyton* rubrum”, “*Trichophyton* interdigitale”, “*Trichophyton* mentagrophytes”, “*Trichophyton* indotineae” “terbinafine”, “antifungal resistance”, “MIC”, “susceptibility”, “prevalence” and combinations of these terms (Table A2). No manual search or snowballing strategy was performed in addition to the predefined electronic database search.

### 4.3. Inclusion and Exclusion Criteria

Studies investigating terbinafine resistance in dermatophytes isolated from human-derived samples, performing species-level differentiation in dermatophytes, published in peer-reviewed journals in English or Turkish, and having the nature of original research were included in the meta-analysis. English and Turkish were selected because these were the languages in which the review team had full proficiency, allowing accurate assessment of study eligibility and data extraction without the need for translation. Eligible studies were required to clearly report the number of terbinafine-resistant isolates and the total number of isolates. Information on antifungal susceptibility testing methods, including MIC methodology and interpretive criteria (CLSI or EUCAST where applicable), was extracted for all included studies. Studies that did not represent routine surveillance populations (Group 3) were included in the overall pooled prevalence analysis but were excluded from the surveillance-based sensitivity analysis.

Case reports, reviews, theses, letters to the editor, and conference abstracts were excluded from the scope of the study. Studies focusing on dermatophytes isolated from animals or environmental sources, not performing species identification, using methods other than standard susceptibility tests accepted by CLSI and EUCAST criteria, and published in languages other than English or Turkish were excluded. Animal-derived isolates were excluded to maintain a human clinical focus. In addition, studies reporting insufficient data that would not allow calculation of resistance prevalence were also excluded.

### 4.4. Study Selection and Data Extraction

Inclusion and exclusion criteria were independently applied in two stages by three authors (I.K., N.I.V and E.P.K.K.). In the first stage, titles and abstracts were read, and then the full texts of the articles selected in the first stage were read. Subsequently, the full texts of the eligible studies were evaluated. Disagreements were resolved by consensus with other researchers (I.H.C. and F.G.H).

From each study; publication year, country, dermatophyte species, total number of isolates, number of terbinafine-resistant isolates, identification method, clinical specimen types, antifungal susceptibility test method, and guideline data used were extracted through a standardized form. Nineteen studies were divided into three groups according to the criteria used to define terbinafine resistance. Group 1 consists of studies determining resistance according to CLSI-based minimum inhibitory concentration (MIC) breakpoints. Group 2 includes studies applying MIC thresholds or epidemiological cut-off values (ECOFFs) defined by EUCAST. Group 3 comprised studies involving selected or enriched populations, such as resistant referrals, treatment-failure isolates, or reference collections, rather than routine surveillance populations. Group 3 studies were included in the overall pooled prevalence estimate but were excluded from the surveillance-based sensitivity analysis to assess the influence of selected or referral-based populations on the pooled estimate.

For each included study, the antifungal susceptibility testing method (CLSI or EUCAST), MIC interpretive criteria, resistance breakpoint or epidemiological cutoff value, and definition of terbinafine resistance were extracted. Because resistance definitions varied across studies, each study was classified according to the original criteria reported by the authors. No attempt was made to reclassify isolates using a unified MIC threshold (Table 1). *SQLE* mutation data were extracted irrespective of whether studies used hotspot PCR or full gene sequencing. Because sequencing coverage and molecular approaches differed substantially among studies, mutation data were summarized descriptively and were not pooled quantitatively.

Studies were temporally divided into two periods as those published in ≤2021 and those published in ≥2022. The main rationale for this division is that terbinafine resistance began to be reported as an “emerging” problem globally in the post-2020 period and a marked increase in the number of publications on this subject has been observed in the literature since 2022. In addition, due to the limited number of studies in the pre-2021 period and the concentration of publications in the post-2022 period, a two period classification was considered a more appropriate and balanced approach for statistical comparisons.

Information regarding species identification methods (ITS sequencing, multiplex PCR, MALDI-TOF MS, sequencing of additional loci, or other molecular methods) was extracted whenever available (Table 1). In several included studies, species-level genotyping or sequence-based confirmation was not performed. Species identification was based on phenotypic or routine laboratory methods as reported by the original authors, and these limitations were not re-evaluated in the present study.

### 4.5. Risk of Bias Assessment

The risk of bias of the included studies were evaluated using the Joanna Briggs Institute (JBI) Prevalence Studies Checklist. Each study was assigned to one of the low, moderate, or high risk categories. Risk of bias assessment was performed independently by two researchers. Those with a JBI assessment score below four were excluded [46]. According to this evaluation, 7 studies were rated as moderate risk and 12 studies as low risk category (Table A4).

### 4.6. Statistical Analysis

This meta-analysis was conducted to evaluate the terbinafine resistance rate in dermatophytes as the primary outcome measure. Pooled prevalence estimates were calculated using a random-effects model, anticipating high heterogeneity among studies.

For variance stabilization of prevalence rates, Freeman–Tukey double arcsine transformation was applied, and results were reported with 95% confidence intervals. Heterogeneity among studies was evaluated with Cochran Q test and I^2^ statistic.

The robustness of the results was tested by sensitivity analyses (leave-one-out) in which individual studies were removed one by one (Figure A1).

Heterogeneity and I^2^ heterogeneity index among the studies included in the meta-analysis were analyzed using CMA (Version 3.0, Biostat, NJ, USA). At the same time, a forest plot was created using R Studio (Version 4.4.2, R Foundation for Statistical Computing, Vienna, Austria). According to the analysis results, a fixed-effect or random-effect model was selected. Forest plots were created to visually summarize individual study results and overall effect sizes. To demonstrate the statistical reliability of antifungal resistance rates reported in the included studies, 95% confidence intervals (CI) were used.

Prevalence data in the studies were evaluated for normality using the Shapiro–Wilk test. Since the distributions did not conform to normal distribution, statistical analysis was performed using non-parametric methods. Differences in terbinafine resistance prevalence among subgroups (study groups, time, *Trichophyton* species, and geographic regions) were evaluated using Kruskal–Wallis and Mann–Whitney U tests. Mean rank values were used to describe the relative distribution of prevalence values among categories. Two-sided *p* < 0.05 was considered statistically significant. Data were analyzed using SPSS software (IBM SPSS Statistics, Version 25.0; IBM Corp., Armonk, NY, USA).

## 5. Conclusions

This meta-analysis shows that terbinafine resistance in dermatophytes has reached a remarkable level worldwide. However, this estimate decreased to 3.2% when analyses were restricted to surveillance-based studies, indicating the influence of selected populations on overall prevalence estimates. Descriptively higher resistance rank values were observed in *T. indotineae* and *T. mentagrophytes*; however, these differences were not statistically significant and should therefore be interpreted with caution. Particularly in the last five years, the high terbinafine MIC values and commonly reported *SQLE* mutations in *Trichophyton* spp. species indicate that dermatophytes should be carefully monitored in terms of resistance dynamics. In addition, it is recommended that laboratory findings be evaluated together with molecular and clinical parameters in the assessment of terbinafine resistance. Increasing resistance reports on a global scale emphasize the need for standardized in vitro susceptibility test methods, clarification of epidemiological cut-off values, and strengthening of molecular surveillance.

## Figures and Tables

**Table 1:** Characteristics of studies included in the meta-analysis [8,9,14,17,18,19,20,21,22,23,24,25,26,27,28,29,30,31,32].

Group	Study	Country	Sample	Identification Method	Species	Method for Detection of Terbinafine Resistance	Guideline	Total Sample	Number of Terbinafine Resistant Strains	Terbinafine Resistance Rate (%)	Cut-Off Values
1	Cañete-Gibas et al., 2023 [17]	North America	Skin samples (including skin scrapings and tissue specimens)	PCR	*T. rubrum*, *T. indotineae*	BMD	CLSI M38 (2022)	271	50	18.45	≥1 µg/mL
1	Nojo et al., 2025 [18]	Japan	Skin and nail samples	Microscopy and culture-based morphological identification	*T. rubrum*, *T. indotineae, T. interdigitale*	BMD	CLSI M38-A2 (2008)	311	10	3.22	≥1 µg/mL
1	Ohara et al., 2025 [19]	Japan	Nail samples	PCR	*T. rubrum*, *T. interdigitale*	BMD	CLSI M38-A2 (2017)	477	17	3.56	≥1 µg/mL
1	Pashootan et al., 2022 [20]	Iran	-	ITS sequencing	*T. rubrum*, *T. indotineae, T. interdigitale*	BMD	CLSI M38-A2 (2008)	123	6	4.88	≥1 µg/mL
1	Taghipour et al., 2020 [21]	Iran	Skin, nail and hair samples	PCR	*T. mentagrophytes*, *T. interdigitale*	BMD	CLSI M38-A2 (2008)	141	5	3.55	≥1 µg/mL
1	Mori et al., 2025 [8]	Japan	Skin and nail samples	PCR	*T. mentagrophytes*, *T. rubrum*, *T. indotineae*, *T. interdigitale*	BMD	CLSI M38-A2 (2017)	701	20	2.85	≥1 µg/mL
2	Moreno-Sabater et al., 2022 [22]	France	Skin and nail samples	PCR	*T. rubrum*, *T. indotineae, T. interdigitale*	BMD	EUCAST E.DEF 11.0 (2021)	580	3	0.52	0.25–0.5 µg/mL
2	Siopi et al., 2021 [23]	Greece	Skin and nail samples	PCR	*T. mentagrophytes*, *T. rubrum*, *T. interdigitale*	BMD	EUCAST E.DEF 11.0 (2021)	112	9	8.04	0.25–0.5 µg/mL
2	Jiang et al., 2021 [24]	China	Skin and hair samples	ITS sequencing	*T. rubrum*	BMD	CLSI M38-A3 (2017)	62	0	0.00	0.25–0.5 µg/mL
2	Kolarczyková et al., 2023 [25]	Czech Republic	Skin and nail samples	PCR	*T. mentagrophytes*, *T. rubrum*	BMD	EUCAST E.DEF 11.0 (2021)	754	6	0.80	>4 mg/L
3	Astvad et al., 2022 [14]	Denmark	-	ITS sequencing	*T. rubrum*, *T. indotineae*	-	EUCAST E.DEF 11.0 (2021)	63	38	60.32	≥1 µg/mL
3	Amin et al., 2024 [9]	India	Skin samples (including skin scrapings)	-	*T. mentagrophytes*, *T. rubrum*	BMD	CLSI M38-A2 (2017)	60	0	0.00	≥1 µg/mL
3	Sardana et al., 2018 [26]	India	Skin samples (including skin scrapings)	Microscopy and culture-based morphological identification	*T. mentagrophytes*, *T. rubrum*	BMD	CLSI M38-A2 (2008)	40	0	0.00	≥1 µg/mL
3	Bhuiyan et al., 2024 [27]	Bangladesh	Skin samples	PCR	*T. mentagrophytes*, *T. rubrum*	In vitro agar-based susceptibility test	EUCAST E.DEF 11.0 (2021)	80	49	61.25	0.125 µg/mL
3	McTaggart et al., 2025 [28]	Canada	-	PCR	*T. indotineae*	BMD	CLSI M38-A2 (2017)	50	36	72.00	≥1 µg/mL
3	De Paepe et al., 2024 [29]	Germany	-	MALDI-TOF MS	*T. indotineae*	BMD	EUCAST E.DEF 11.0 (2022)	20	9	45.00	≥0.5 µg/mL
3	Bidaud et al., 2023 [30]	France	-	ITS sequencing	*T. indotineae*, *T. interdigitale*	BMD	EUCAST E.DEF (2021)	79	10	12.66	0.25–0.5 µg/mL
3	Shankarnarayan et al., 2023 [31]	India	Skin samples (including skin scrapings)	PCR	*T. mentagrophytes*, *T. rubrum*	-	CLSI M38-A2 (2008)	136	15	11.03	≥1 µg/mL
3	Oguz et al., 2024 [32]	Türkiye	Skin, nail and hair samples	Microscopy and culture-based morphological identification	*T. mentagrophytes*, *T. rubrum*	BMD	CLSI	51	14	27.45	≥0.5 µg/mL

**Table 2:** Pooled prevalence estimates and heterogeneity statistics for terbinafine resistance.

Model		Fixed	Random
**Effect size and 95% interval**	Number Studies	10	10
	Point estimate	0.060	0.032
	Lower limit	0.050	0.015
	Upper limit	0.071	0.067
**Test of null (2-Tail)**	Z-value	−29.532	−8.564
	*p*-value	0.000	0.000
**Heterogeneity**	Q-value	135.085	
	df (Q)	9	
	*p*-value	0.000	
	i^2^	93.338	
**Tau-squared**	Tau^2^	1.370	
	Standard error	0.890	

## Data Availability

The original contributions presented in this study are included in the article. Further inquiries can be directed to the corresponding author.

## Appendix A

**Table A1:** Prisma checklist.

Section and Topic	Item #	Checklist Item	Location Where Item Is Reported
TITLE			
Title	1	Identify the report as a systematic review.	1
ABSTRACT			
Abstract	2	See the PRISMA 2020 for Abstracts checklist.	1
INTRODUCTION			
Rationale	3	Describe the rationale for the review in the context of existing knowledge.	2–3
Objectives	4	Provide an explicit statement of the objective(s) or question(s) the review addresses.	3
METHODS			
Eligibility criteria	5	Specify the inclusion and exclusion criteria for the review and how studies were grouped for the syntheses.	4–6
Information sources	6	Specify all databases, registers, websites, organizations, reference lists and other sources searched or consulted to identify studies. Specify the date when each source was last searched or consulted.	4–6
Search strategy	7	Present the full search strategies for all databases, registers and websites, including any filters and limits used.	4–6
Selection process	8	Specify the methods used to decide whether a study met the inclusion criteria of the review, including how many reviewers screened each record and each report retrieved, whether they worked independently, and if applicable, details of automation tools used in the process.	4–6
Data collection process	9	Specify the methods used to collect data from reports, including how many reviewers collected data from each report, whether they worked independently, any processes for obtaining or confirming data from study investigators, and if applicable, details of automation tools used in the process.	4–6
Data items	10a	List and define all outcomes for which data were sought. Specify whether all results that were compatible with each outcome domain in each study were sought (e.g., for all measures, time points, analyses), and if not, the methods used to decide which results to collect.	4–6
	10b	List and define all other variables for which data were sought (e.g., participant and intervention characteristics, funding sources). Describe any assumptions made about any missing or unclear information.	4–6
Study risk of bias assessment	11	Specify the methods used to assess risk of bias in the included studies, including details of the tool(s) used, how many reviewers assessed each study and whether they worked independently, and if applicable, details of automation tools used in the process.	4–6
Effect measures	12	Specify for each outcome the effect measure(s) (e.g., risk ratio, mean difference) used in the synthesis or presentation of results.	4–6
Synthesis methods	13a	Describe the processes used to decide which studies were eligible for each synthesis (e.g., tabulating the study intervention characteristics and comparing against the planned groups for each synthesis (item #5)).	4
	13b	Describe any methods required to prepare the data for presentation or synthesis, such as handling of missing summary statistics, or data conversions.	4
	13c	Describe any methods used to tabulate or visually display results of individual studies and syntheses.	4
	13d	Describe any methods used to synthesize results and provide a rationale for the choice(s). If meta-analysis was performed, describe the model(s), method(s) to identify the presence and extent of statistical heterogeneity, and software package(s) used.	3, 4
	13e	Describe any methods used to explore possible causes of heterogeneity among study results (e.g., subgroup analysis, meta-regression).	4
	13f	Describe any sensitivity analyses conducted to assess robustness of the synthesized results.	4
Reporting bias assessment	14	Describe any methods used to assess risk of bias due to missing results in a synthesis (arising from reporting biases).	4–6
Certainty assessment	15	Describe any methods used to assess certainty (or confidence) in the body of evidence for an outcome.	4–6
RESULTS			
Study selection	16a	Describe the results of the search and selection process, from the number of records identified in the search to the number of studies included in the review, ideally using a flow diagram.	7–15
	16b	Cite studies that might appear to meet the inclusion criteria, but which were excluded, and explain why they were excluded.	7–15
Study characteristics	17	Cite each included study and present its characteristics.	7–15
Risk of bias in studies	18	Present assessments of risk of bias for each included study.	7–15
Results of individual studies	19	For all outcomes, present, for each study: (a) summary statistics for each group (where appropriate) and (b) an effect estimate and its precision (e.g., confidence/credible interval), ideally using structured tables or plots.	7–15
Results of syntheses	20a	For each synthesis, briefly summarize the characteristics and risk of bias among contributing studies.	7–15
	20b	Present results of all statistical syntheses conducted. If meta-analysis was done, present for each the summary estimate and its precision (e.g., confidence/credible interval) and measures of statistical heterogeneity. If comparing groups, describe the direction of the effect.	7–15
	20c	Present results of all investigations of possible causes of heterogeneity among study results.	7–15
	20d	Present results of all sensitivity analyses conducted to assess the robustness of the synthesized results.	7–15
Reporting biases	21	Present assessments of risk of bias due to missing results (arising from reporting biases) for each synthesis assessed.	16–18
Certainty of evidence	22	Present assessments of certainty (or confidence) in the body of evidence for each outcome assessed.	16–18
DISCUSSION			
Discussion	23a	Provide a general interpretation of the results in the context of other evidence.	16–18
	23b	Discuss any limitations of the evidence included in the review.	16–18
	23c	Discuss any limitations of the review processes used.	16–18
	23d	Discuss implications of the results for practice, policy, and future research.	16–18
OTHER INFORMATION			
Registration and protocol	24a	Provide registration information for the review, including register name and registration number, or state that the review was not registered.	1, 4
	24b	Indicate where the review protocol can be accessed, or state that a protocol was not prepared.	1, 4
	24c	Describe and explain any amendments to information provided at registration or in the protocol.	1, 4
Support	25	Describe sources of financial or non-financial support for the review, and the role of the funders or sponsors in the review.	-
Competing interests	26	Declare any competing interests of review authors.	13
Availability of data, code and other materials	27	Report which of the following are publicly available and where they can be found: template data collection forms; data extracted from included studies; data used for all analyses; analytic code; any other materials used in the review.	13

**Table A2:** Search terms.

Database	Search Terms
PubMed/MEDLINE	(“Dermatophytes”[Mesh] OR dermatophyte* OR “*Trichophyton*”[Mesh] OR “*Trichophyton rubrum*” OR “*Trichophyton interdigitale*” OR “*Trichophyton mentagrophytes*” OR “*Trichophyton indotineae*”) AND (“Terbinafine”[Mesh] OR terbinafine) AND (“Drug Resistance, Fungal”[Mesh] OR resistance OR “antifungal resistance” OR susceptibility OR MIC OR “minimum inhibitory concentration”) AND (prevalence OR epidemiology)
Scopus	TITLE-ABS-KEY (dermatophyte* OR “*Trichophyton* rubrum” OR “*Trichophyton interdigitale*” OR “*Trichophyton mentagrophytes*” OR “*Trichophyton indotineae*”) AND TITLE-ABS-KEY (terbinafine) AND TITLE-ABS-KEY (“antifungal resistance” OR resistance OR susceptibility OR MIC OR prevalence)
Web of Science	TS = (dermatophyte* OR “*Trichophyton rubrum*” OR “*Trichophyton interdigitale*” OR “*Trichophyton mentagrophytes*” OR “*Trichophyton indotineae*”) AND TS = (terbinafine) AND TS = (“antifungal resistance” OR resistance OR susceptibility OR MIC OR prevalence)
Embase	(‘dermatophyte’/exp OR dermatophyte*:ti,ab OR ‘*Trichophyton*’/exp OR “*Trichophyton rubrum*”:ti,ab OR “*Trichophyton interdigitale*”:ti,ab OR “*Trichophyton mentagrophytes*”:ti,ab OR “*Trichophyton indotineae*”:ti,ab) AND (‘terbinafine’/exp OR terbinafine:ti,ab) AND (‘antifungal resistance’/exp OR resistance:ti,ab OR susceptibility:ti,ab OR MIC:ti,ab OR prevalence:ti,ab)

(*) denotes truncation and was used to retrieve all possible word endings for the root term where applicable.

**Table A3:** Species-level data extraction for terbinafin resistance analysis in dermatophytes.

Group	Study	Species	Total Sample	Number of Terbinafine Resistant Strains	Terbinafine Resistance Rate (%)
1	Cañete-Gibas et al., 2023 [17]	*T. rubrum*	117	21	17.95
1	Cañete-Gibas et al., 2023 [17]	*T. indotineae*	21	21	100.00
1	Nojo et al., 2025 [18]	*T. rubrum*	188	9	4.79
1	Nojo et al., 2025 [18]	*T. indotineae*	1	0	0.00
1	Nojo et al., 2025 [18]	*T. interdigitale*	122	1	0.82
1	Ohara et al., 2025 [19]	*T. rubrum*	353	16	4.53
1	Ohara et al., 2025 [19]	*T. interdigitale*	82	1	1.22
1	Pashootan et al., 2022 [20]	*T. rubrum*	15	0	0.00
1	Pashootan et al., 2022 [20]	*T. indotineae*	10	6	60.00
1	Pashootan et al., 2022 [20]	*T. interdigitale*	28	0	0.00
1	Taghipour et al., 2020 [21]	*T. mentagrophytes*	45	5	11.11
1	Taghipour et al., 2020 [21]	*T. interdigitale*	96	0	0.00
1	Mori et al., 2025 [8]	*T. mentagrophytes*	199	1	0.50
1	Mori et al., 2025 [8]	*T. rubrum*	16	16	100.00
1	Mori et al., 2025 [8]	*T. indotineae*	2	1	50.00
1	Mori et al., 2025 [8]	*T. interdigitale*	119	2	1.68
2	Moreno-Sabater et al., 2022 [22]	*T. rubrum*	436	1	0.23
2	Moreno-Sabater et al., 2022 [22]	*T. indotineae*	136	1	0.74
2	Moreno-Sabater et al., 2022 [22]	*T. interdigitale*	8	1	12.50
2	Siopi et al., 2021 [23]	*T. mentagrophytes*	24	9	37.50
2	Siopi et al., 2021 [23]	*T. rubrum*	70	0	0.00
2	Siopi et al., 2021 [23]	*T. interdigitale*	12	0	0.00
2	Jiang et al., 2021 [24]	*T. rubrum*	62	0	0.00
2	Kolarczyková et al., 2023 [25]	*T. mentagrophytes*	240	6	2.50
2	Kolarczyková et al., 2023 [25]	*T. rubrum*	514	0	0.00

**Table A4:** JBI Critical Appraisal Checklist for studies.

Studies	1. Was the Sample Frame Appropriate to Address the Target Population?	2. Were Study Participants Sampled in an Appropriate Way?	3. Was the Sample Size Adequate?	4. Were the Study Subjects and the Setting Described in Detail?	5. Was the Data Analysis Conducted with Sufficient Coverage of the Identified Sample?	6. Were Valid Methods Used for the Identification of the Condition?	7. Was the Condition Measured in a Standard, Reliable Way for All Participants?	8. Was There Appropriate Statistical Analysis?	9. Was the Response Rate Adequate, and If Not, Was the Low Response Rate Managed Appropriately?	General Risk
Cañete-Gibas et al., 2023 [17]	Y	Y	Y	Y	U	Y	Y	Y	NA	Low
Nojo et al., 2025 [18]	Y	Y	Y	Y	Y	Y	Y	N	NA	Moderate
Ohara et al., 2025 [19]	Y	U	Y	Y	Y	Y	Y	N	NA	Moderate
Pashootan et al., 2022 [20]	Y	U	Y	Y	Y	Y	Y	Y	NA	Low
Taghipour et al., 2020 [21]	Y	U	Y	Y	Y	Y	Y	N	NA	Moderate
Mori et al., 2025 [8]	Y	U	Y	Y	Y	Y	Y	Y	NA	Low
Moreno-Sabater et al., 2022 [22]	Y	Y	Y	Y	Y	Y	Y	N	NA	Low
Siopi et al., 2021 [23]	Y	U	Y	Y	Y	Y	Y	Y	NA	Low
Jiang et al., 2021 [24]	Y	U	Y	Y	Y	Y	Y	Y	NA	Low
Kolarczyková et al., 2023 [25]	Y	Y	Y	Y	Y	Y	Y	N	NA	Low
Astvad et al., 2022 [14]	N	N	U	Y	U	Y	Y	Y	NA	Moderate
Amin et al., 2024 [9]	N	Y	Y	Y	Y	Y	Y	N	NA	Moderate
Sardana et al., 2018 [26]	N	Y	U	Y	Y	Y	Y	N	NA	Moderate
Bhuiyan et al., 2024 [27]	N	N	Y	Y	Y	Y	Y	N	NA	Moderate
McTaggart et al., 2025 [28]	N	N	Y	Y	Y	Y	Y	N	NA	Moderate
De Paepe et al., 2024 [29]	N	N	N	Y	Y	Y	Y	N	NA	Moderate
Bidaud et al., 2023 [30]	N	N	Y	Y	Y	Y	Y	N	NA	Moderate
Shankarnarayan et al., 2023 [31]	N	U	Y	Y	Y	Y	Y	N	NA	Moderate
Oguz et al., 2024 [32]	N	N	U	Y	Y	U	Y	Y	NA	Moderate

Y: Yes, N: No, U: Unclear, NA: Not applicable.
